# Control of lifespan and survival by *Drosophila* NF-κB signaling through neuroendocrine cells and neuroblasts

**DOI:** 10.18632/aging.104196

**Published:** 2020-11-24

**Authors:** Sinan Khor, Dongsheng Cai

**Affiliations:** 1 Department of Molecular Pharmacology, Albert Einstein College of Medicine, Bronx, NY 10461, USA; 2 Diabetes Research Center, Albert Einstein College of Medicine, Bronx, NY 10461, USA; 3 Institute for Aging Research, Albert Einstein College of Medicine, Bronx, NY 10461, USA

**Keywords:** aging, brain, neuron, *Drosophila*

## Abstract

We report a comparative analysis of the effects of immune activation in the fly nervous system using genetic activation models to target *Drosophila* NF-κB within Toll versus Imd pathways. Genetic gain-of-function models for either pathway pan-neuronally as well as in discrete subsets of neural cells including neuroendocrine insulin-producing cells (IPCs) or neuroblasts reduce fly lifespan, however, these phenotypes in IPCs and neuroblasts are stronger with Toll activation than Imd activation. Of note, while aging is influenced more by Toll/NF-κB activation in IPCs during adulthood, neuroblasts influence aging more substantially during development. The study then focused on Toll/NF-κB inhibition, revealing that IPCs or neuroblasts are important for the effects of lifespan and healthspan extension but in a life stage-dependent manner while some of these effects display sexual dimorphism. Importantly, co-inhibition of Toll/NF-κB pathway in IPCs and neuroblasts increased fly lifespan greater than either cell population, suggesting that independent mechanisms might exist. Toll/NF-κB inhibition in IPCs was also sufficient to enhance survival under various fatal stresses, supporting the additional benefits to fly healthspan. In conclusion, IPCs and neuroblasts are important for *Drosophila* NF-κB for controlling lifespan.

## INTRODUCTION

As an organism ages, it undergoes various physiological changes that lead to breakdown and loss of tissue function and eventual death. The mechanisms that govern the process of aging have slowly been delineated through studies in various animal models. Initial studies in the nematode *Caenorhabditis elegans* and fruit fly *Drosophila melanogaster* have shown that neurons or subsets of neurons can mediate aging progression and lifespan [1–5]. In mammals, the hypothalamus has been shown to be important for the control of whole-body aging and the underlying molecular pathways include pro-inflammatory nuclear factor kappa B (NF-κB) signaling [6, 7]. The pars intercerebralis (PI) is a subregion of the *Drosophila* nervous system that contains insulin-producing cells (IPCs), which exhibit similarity to the neuroendocrine cells of the mammalian hypothalamus [8, 9]. These neurosecretory cells primarily function through an endocrine mechanism leading to the production of insulin-like peptides (Dilps) that are secreted into systemic circulation to activate insulin signaling in peripheral target tissues [9]. Several studies have examined how Dilps from the brain, Dilp2, Dilp3, and Dilp5, and IPCs themselves, regulate the lifespan of *Drosophila* through Dilp gene targeting or direct ablation of the IPCs [5, 10–13]. However, no efforts have been made to investigate the aging relevance of NF-κB signaling in these neuroendocrine cells of *Drosophila*.

In addition to hypothalamic endocrine cells, the importance of hypothalamic neural stem cells (htNSC) for aging and survival was also recently recognized in rodents [7, 14, 15]. The nervous system of *Drosophila* contains neuroblasts, which exhibit similarities to vertebrate neural stem cells and give rise to the larval and adult nervous system [16, 17]. During the developmental stages of the *Drosophila* life cycle, neuroblasts give rise to major cellular components of the larval and adult nervous systems, including neurons and glial cells. Interestingly, unlike in rodents in which neural stem cells exist in adult brain, *Drosophila* neuroblasts are thought to function exclusively in development, ceasing activity within the first week or so of adult life [18, 19], although some limited evidence suggests a possible existence of adult neurogenesis in response to stress or injury [20, 21]. However, no research has been documented to study the aging relevance of NF-κB signaling in neuroblasts of *Drosophila*.

*Drosophila* possess two NF-κB signaling pathways that are uniquely active against different pathogens, the Toll signaling pathway, which is activated in response to most gram-positive bacterial and fungal infections, and the immunodeficient (Imd) signaling pathway, which is activated against most gram-negative infections [22, 23]. Through transmembrane receptors, each pathway activates a signaling cascade to activate distinct NF-κB transcription factors that regulate expression of a plethora of target genes including the production of antimicrobial peptides (AMPs). The Toll pathway activates the transcription factors Dorsal and Dif through degradation of the IκB-like inhibitor Cactus [22], while the Imd pathway activates the transcription factor Relish through cleavage of its internal inhibitory domain [23]. Studies have shown the pathways to possess levels of distinction in the target genes regulated, but crosstalk between them has also been demonstrated and appreciated [24, 25]. In this study, we systematically profiled different *Drosophila* NF-κB signaling pathways and different neural cell types in terms of influences on lifespan in normal physiology. Our observations highlight the importance of neuroendocrine cells and neuroblasts for controlling lifespan through NF-κB and the effectiveness of suppressing Toll pathway in both cell types in increasing fly lifespan. Our study further extended to reveal the additional benefits of Toll pathway inhibition in neuroendocrine cells in counteracting fatal stresses leading to enhanced survival.

## RESULTS

### Pan-neuronal Toll/NF-κB pathway activation dramatically reduces lifespan

**Figure 1:**
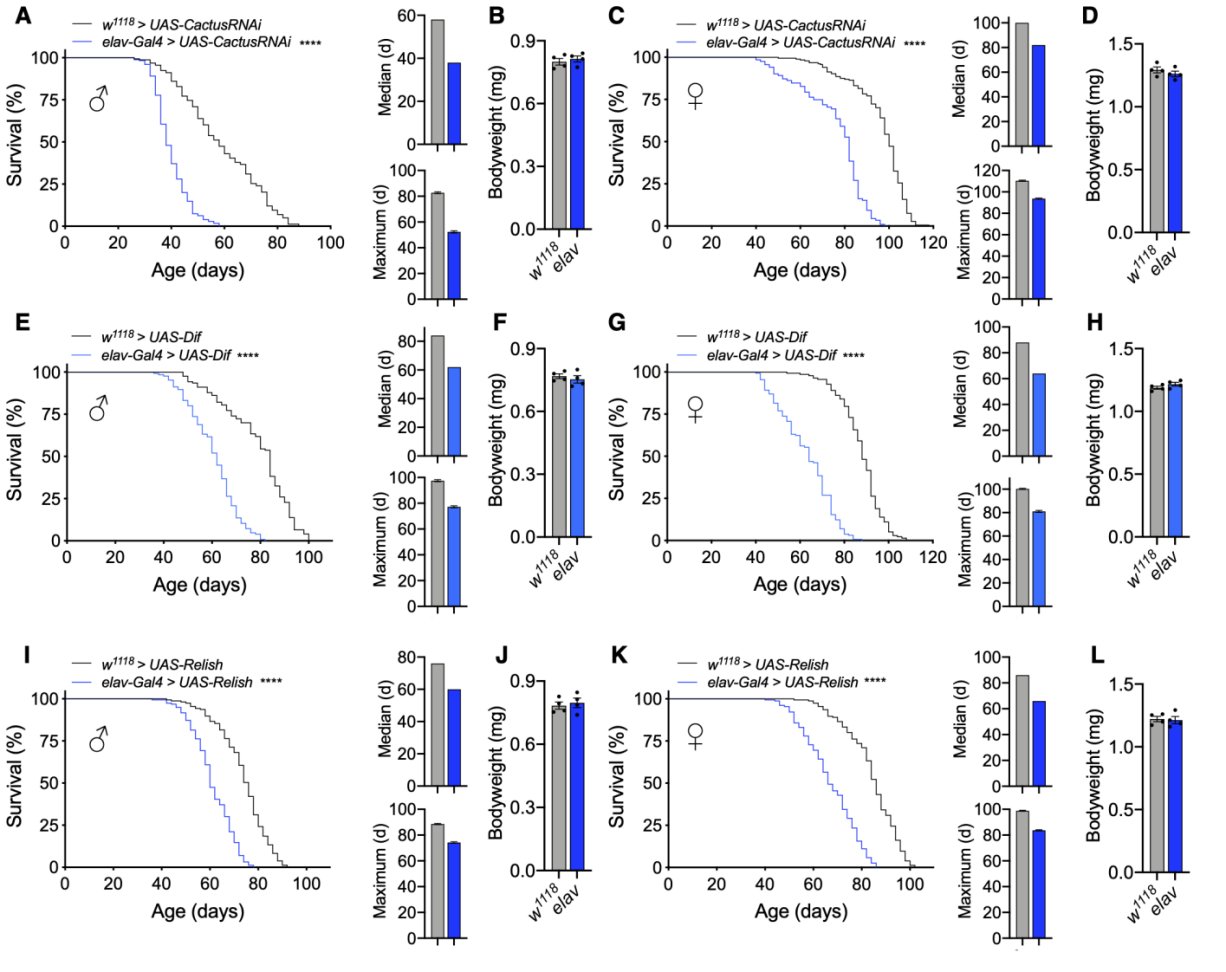
**Pan-neuronal activation of Toll or Imd pathway signaling shortens lifespan.** (**A**–**D**) Survival, median lifespan, and 10% max lifespan (**A**) and bodyweight (**B**) of *elav-Gal4/UAS-CactusRNAi* males (blue) and *UAS-CactusRNAi/+* (black) control males. Corresponding lifespan analysis (**C**) and bodyweight (**D**) for females. (**E**–**H**) Survival, median lifespan, and 10% max lifespan (**E**) and bodyweight (**F**) of *elav-Gal4/UAS-Dif* males (blue) and *UAS-Dif/+* (black) control males. Corresponding lifespan analysis (**G**) and bodyweight (**H**) for females. (**I**–**L**) Survival, median lifespan, and 10% max lifespan (**I**) and bodyweight (**J**) of *UAS-Relish/+;elav-Gal4/+* males (blue) and *UAS-Relish/+;+/+* (black) control males. Corresponding lifespan analysis (**K**) and bodyweight (**L**) for females. Data information: statistics for curve comparisons are shown in the figure. Error bars represent mean ± SEM. **** p<.0001 (log-rank test). n = at least 100 flies for each genotype in lifespan experiments. n = 4 vials of 15 flies for each genotype for bodyweight measurements.

To assess how *Drosophila* NF-κB signaling in the nervous system impacts aging, we employed the Gal4/UAS system and assessed different genetic gain-of-function models to increase Toll or Imd pathway activation [26]. The Toll signaling pathway converges on the activation of two NF-κB transcription factors, Dorsal and Dif, that are normally held inactive in the cytoplasm bound to Cactus, the homolog of mammalian IκB [22]. To activate Toll signaling, we knocked down *Cactus* pan-neuronally using the Gal4 driver *elav-Gal4*, which is expressed in neurons. The *Cactus* RNAi strain (*y^1^,v^1^,sc;;UAS-CactusRNAi*) was in a genetic background of *y^1^,v^1^,sc*, with identifying markers of yellow body and vermillion eye color, while *elav-Gal4* was in the *w^1118^* genetic background with the identifying marker of red eye color. We initially evaluated all possible controls, including the pure background of *UAS-CactusRNAi* and the mixed background of *UAS-CactusRNAi* crossed to *w^1118^*strain (*w^1118^ > y^1^,v^1^,sc;;UAS-CactusRNAi)*, confirming that *UAS-CactusRNAi* did not significantly affect lifespan of flies in either case. We further confirmed that *elav-Gal4* did not affect lifespan, showing that the lifespan of *elav-Gal4* flies (*w^1118^ > w^1118^;;elav-Gal4)* was comparable to that of *w^1118^* flies (*w^1118^ > w^1118^)* (Supplementary Figure 1A, 1B). The lifespan of *w^1118^ > y^1^,v^1^,sc;;UAS-CactusRNAi* flies did differ from *w^1118^* lifespan (Supplementary Figure 1A, 1B and Figure 1A, 1C), which was unsurprising given that *Drosophila* lifespan is sensitive to different genetic backgrounds [27, 28]. Thus, it was important for us to employ genetically matched experimental controls (*w^1118^ > UAS-CactusRNAi)* for analyzing the lifespan of *elav-Gal4 > UAS-CactusRNAi* model. We then analyzed the effect of *Cactus* knockdown pan-neuronally and observed a dramatic reduction in the lifespan of males and females (Figure 1A, 1C). Given that body size can be positively or negatively correlated with lifespan in flies [29], we measured their body weight and verified that offspring were similar sized (Figure 1B, 1D), suggesting that lifespan reduction was not importantly due to a developmental defect. We also examined another independent line of *UAS-CactusRNAi* crossed with *elev-Gal4* and have confirmed the same phenotype of lifespan loss in the absence of body weight change.

As discussed above, upon activation, the Toll pathway signals to degrade Cactus, freeing the NF-κB transcription factors Dorsal and Dif to dimerize and undergo nuclear translocation. To further verify this to be an effect due to Toll pathway activation, we utilized a different genetic model to increase Toll signaling through *Dif* overexpression (*UAS-Dif*). We chose to examine Dif given that it is the major transcription factor for Toll signaling during the adult stage comparative to development, in which Dorsal is more influential [22]. Using this model, we consistently found a dramatic reduction in the lifespan of male and female flies with pan-neuronal *Dif* overexpression (*elav-Gal4 > UAS-Dif*) comparative to the genetic background-matched control flies (*w^1118^ > UAS-Dif*) (Figure 1E, 1G) without change in bodyweight (Figure 1F, 1H), which corroborated our *Cactus* knockdown findings. To verify that these effects were not due to a developmental defect, we assessed the motor function of young flies as a measure of fitness by negative geotaxis and did not observe gross motor deficits in young flies of either cross (Supplementary Figure 1C, 1D). Because the *UAS-Dif* strain was in a *w^1118^* background, in conjunction with the *UAS-CactusRNAi* model presented above, we provided two independent cases under two different genetic backgrounds (the mixed background with *Cactus* knockdown and a homogenous genetic background with *Dif* overexpression) showing the same effect of lifespan loss.

We comparatively examined the Imd pathway, the other NF-κB pathway which has been implicated in aging and neurodegeneration [30–32]. To assess this, we found that pan-neuronal *Relish* overexpression (*elav-Gal4 > UAS-Relish*) indeed displayed a reduction in lifespan compared to control flies (*w^1118^ > UAS-Relish*) (Figure 1I, 1K) without affecting body weight (Figure 1J, 1L). However, the magnitude of lifespan loss due to Imd activation was smaller compared than the effect of Toll pathway activation. For curiosity, we also tried to maximally increase Imd pathway activation by using a construct expressing only the constitutively-active Rel domain of Relish (*UAS-Rel*). Using this system, we found that *Rel* overexpression (*elav-Gal4 > UAS-Rel*) increased the extent of lifespan loss (Supplementary Figure 2), although we did not further address if such maximal Imd activation could lead to a cross-over effect for Toll activation. Taken together, these results indicate that Toll or Imd pathway activation in neurons can both negatively affect fly lifespan, and the Toll pathway plays a more sensitive role in this effect.

### Toll/NF-κB activation in IPCs or neuroblasts sufficiently leads to lifespan loss

**Figure 2:**
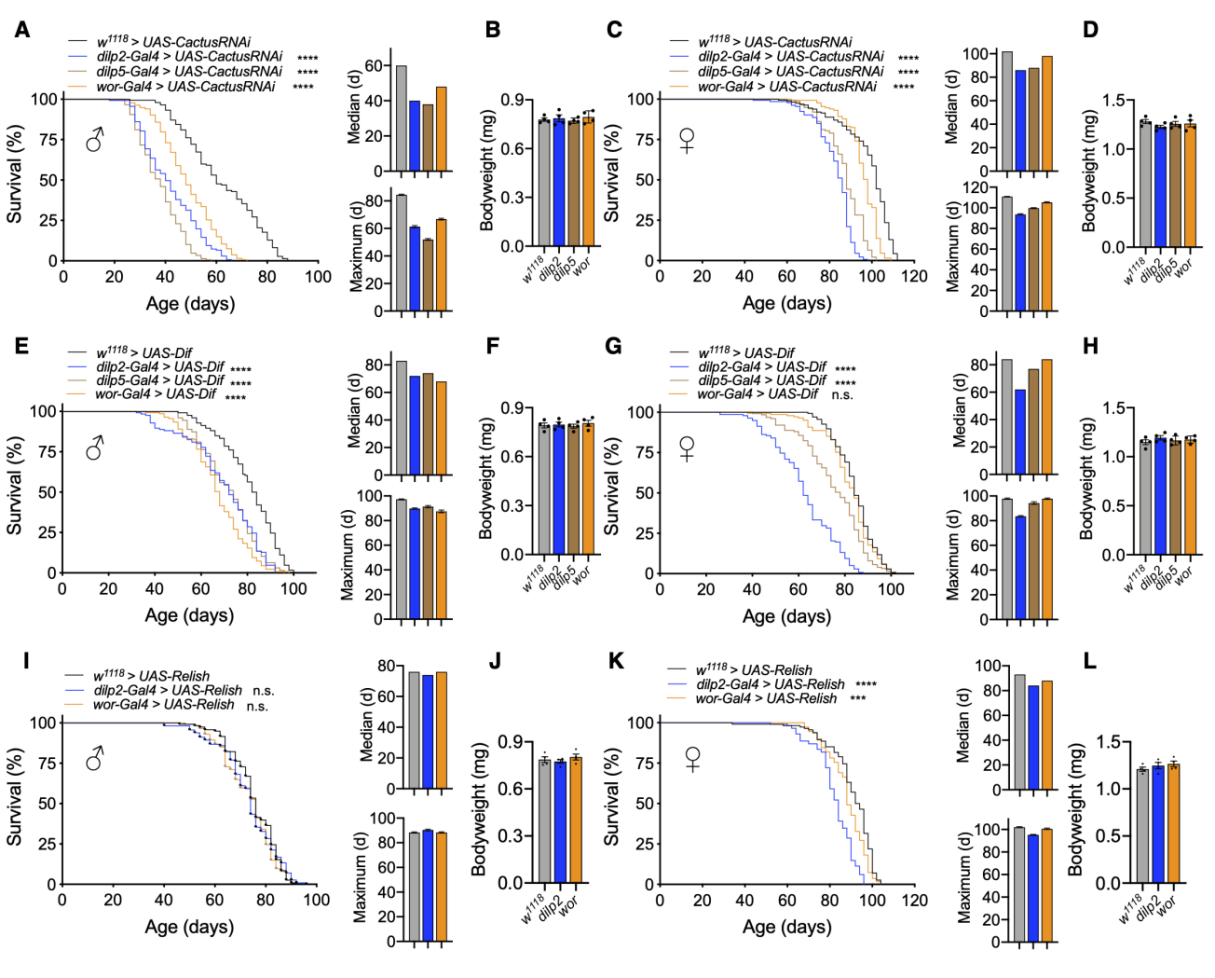
**Activation of Toll pathway signaling in IPCs or neuroblasts shortens lifespan.** (**A**–**D**) Survival, median lifespan, and 10% max lifespan (**A**) and bodyweight (**B**) of *dilp2-Gal4/+;UAS-CactusRNAi/+* males (blue), *dilp5-Gal4/+;UAS-CactusRNAi/+* males (brown), *wor-Gal4/+;UAS-CactusRNAi/+* males (orange), and +/+;*UAS-CactusRNAi/+* (black) control males. Corresponding lifespan analysis (**C**) and bodyweight (**D**) for females. (**E**–**H**) Survival, median lifespan, and 10% max lifespan (**E**) and bodyweight (**F**) of *dilp2-Gal4/+;UAS-Dif*/+ males (blue), *dilp5-Gal4/+;UAS-Dif /+* males (brown), *wor-Gal4/+;UAS-Dif/+* males (orange), and +/+;*UAS-Dif/+* (black) control males. Corresponding lifespan analysis (**G**) and bodyweight (**H**) for females. (**I**–**L**) Survival, median lifespan, and 10% max lifespan (**I**) and bodyweight (**J**) of *dilp2-Gal4/UAS-Relish* males (blue), *wor-Gal4/UAS-Relish* males (orange), and *UAS-Relish/+* (black) control males. Corresponding lifespan analysis (**K**) and bodyweight (**L**) for females. Data information: statistics for curve comparisons are shown in the figure. Error bars represent mean ± SEM. *** p<.001, **** p<.0001, n.s. not significant (log-rank test). n = at least 100 flies for each genotype in lifespan experiments. n = 4 vials of 15 flies for each genotype for bodyweight measurements.

Given that pan-neuronal Toll activation reduced fly lifespan, we wanted to determine if targeting innate immune activation in subsets of cells in the nervous system was impactful on fly lifespan. Prior work in rodent models had indicated the significance of NF-κB activation in the hypothalamus or in neural stem cells as a mechanistic component of aging progression [6, 7], and we sought to test if this could be evolutionarily conserved. Of interest to us were the brain IPCs in the pars intercerebralis, which synthesize insulin-like peptides and are functionally homologous to the hypothalamus and neuroblasts, which are equivalent to mammalian neural stem cells. Using our model for Cactus knockdown, we targeted RNAi expression using the *dilp2-Gal4* and *dilp5-Gal4* drivers, which are expressed in IPCs, as well as the *wor-Gal4* driver, which is expressed in neuroblasts. Although some Dilp5 peptide can be found outside of IPCs such as the ovaries and renal tubules, *dilp5-Gal4* has been confirmed to be present most exclusively in the nervous system [33]. Due to differences in genetic background of the *UAS-CactusRNAi* and *Gal4* lines, we verified that our Gal4 drivers did not influence lifespan (Supplementary Figure 3A, 3B). Next, we found that IPC-specific *Cactus* knockdown (*dilp2-Gal4 > UAS-CactusRNAi* and *dilp5-Gal4 > UAS-CactusRNAi*) greatly reduced lifespan of male and female flies compared to genetically-matched control flies (*w^1118^ > UAS-CactusRNAi*) (Figure 2A, 2C). Since reduced insulin signaling has been shown to alter body size [12, 34], we examined the body weight of these flies but did not see any appreciable change in body weight (Figure 2B, 2D), suggesting that importantly, the phenotype of lifespan loss was not due to a developmental issue. Further examination of IPC-specific *dilp* expression revealed a modest reduction in *dilp2* and *dilp5* expression using *dilp5-Gal4* but not with *dilp2-Gal4* (data not shown), suggesting the effect of Toll pathway activation on aging was likely not due to changes in *dilp* expression. Apart from IPC-specific effects, *Cactus* knockdown in neuroblasts (*wor-Gal4 > UAS-CactusRNAi*) influenced the lifespan of male flies more so than female flies, suggesting a possible sex-specific effect on lifespan (Figure 2A, 2C).

To further verify that Toll signaling activation was responsible for this effect, we overexpressed Dif in IPCs (*dilp2-Gal4 > UAS-Dif* and *dilp5-Gal4 > UAS-Dif)* or neuroblasts (*wor-Gal4 > UAS-Dif*) and observed similar effects (Figure 2E–2H). Neuroblast-specific *Dif* overexpression recapitulated our observations with *Cactus* knockdown, showing a stronger effect in males than in females (Figure 2E, 2G). These results further support that Toll gain-of-function in IPCs and neuroblasts was stimulatory for fly aging, and neuroblast Toll signaling affected aging in males more than females. For comparison, we also targeted the Imd pathway to see if these effects were specific to Toll pathway gain-of-function models. To do so, we overexpressed *Relish* in IPCs (*dilp2-Gal4 > UAS-Relish*) or neuroblasts (*wor-Gal4 > UAS-Relish*). We observed only marginal changes in the lifespan of male and female flies with *Relish* overexpression in IPCs or neuroblasts compared to control flies (*w^1118^ > UAS-Relish*) (Figure 2I–2L). On the other hand, overexpression of the constitutively-active *Rel* domain of Relish in IPCs (*dilp2-Gal4 > UAS-Rel* and *dilp5-Gal4 > UAS-Rel*) or neuroblasts (*wor-Gal4 > UAS-Rel*) led to an increase in lifespan loss compared to control flies (*w^1118^ > UAS-Rel*) (Supplementary Figure 3C, 3D). Taken together, NF-κB activation in IPCs or neuroblasts reduces lifespan, with the Toll pathway being more responsible for this effect than the Imd pathway.

### Toll/NF-κB in IPCs versus neuroblasts controls lifespan in different life stages

**Figure 3:**
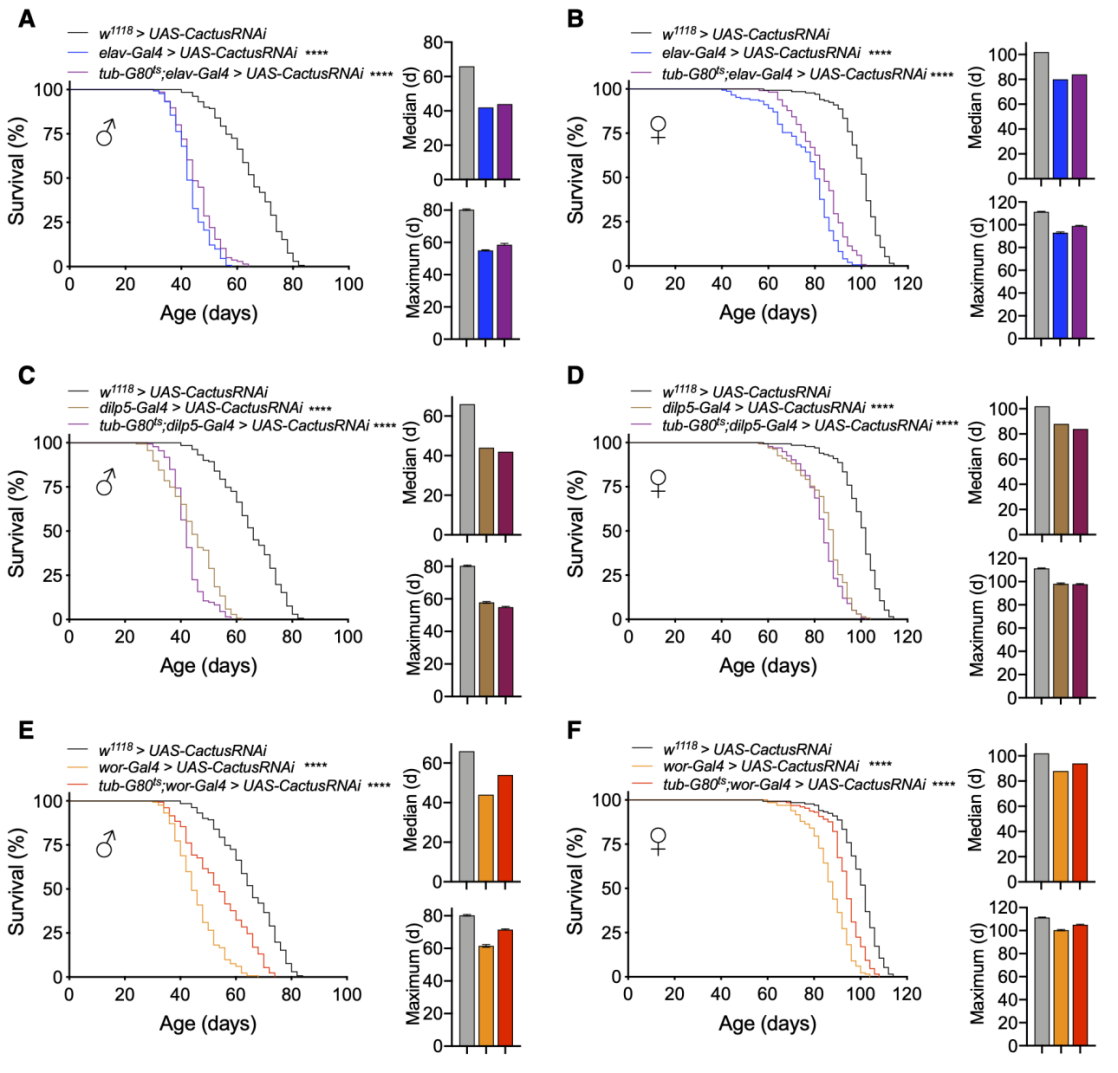
**Lifespan effects of Toll pathway activation in IPCs and neuroblasts are stage-dependent.** (**A**, **B**) Survival, median lifespan, and 10% max lifespan of *elav-Gal4/UAS-CactusRNAi* males (blue), *tub-Gal80^ts^/+;elav-Gal4/UAS-CactusRNAi* males (purple), and *+/+;UAS-CactusRNAi/+* (black) control males (**A**). Corresponding lifespan analysis for females (**B**). (**C**, **D**) Survival, median lifespan, and 10% max lifespan of *dilp5-Gal4/+;UAS-CactusRNAi/+* males (brown), *dilp5-Gal4/+;tub-Gal80^ts^/UAS-CactusRNAi* males (purple), and *+/+;UAS-CactusRNAi/+* (black) control males (**C**). Corresponding lifespan analysis for females (**D**). (**E**, **F**) Survival, median lifespan, and 10% max lifespan of *wor-Gal4/+;UAS-CactusRNAi/+* males (orange), *wor-Gal4/+;tub-Gal80^ts^/UAS-CactusRNAi* males (red), and *+/+;UAS-CactusRNAi/+* (black) control males (**E**). Corresponding lifespan analysis for females (**F**). Data information: statistics for curve comparisons are shown in the figure. Error bars represent mean ± SEM. **** p<.0001 (log-rank test). n = at least 100 flies for each genotype in lifespan experiments.

Because the lifespan loss with Toll pathway activation in IPCs or neuroblasts was stronger than with Imd pathway activation, we focused on Toll pathway models for subsequent experiments. Given that *elav-Gal4* is expressed in neurons from embryonic stage 12 onward [35] and IPC and neuroblast drivers are expressed developmentally [36, 37], we developed conditional genetic fly models of adult manipulations to test if the reduction in lifespan by Toll pathway activation was due to developmental changes or adult-specific effects. To do so, we utilized a temperature-sensitive Gal80 (*tub-Gal80^ts^*) to conditionally knockdown *Cactus* in adult flies by breeding flies during development at 18° C and transferring to 25° C after eclosion. We first tested our *elav-Gal4* model and observed that pan-neuronal adult-specific *Cactus* knockdown (*tub-Gal80^ts^;elav-Gal4 > UAS-CactusRNAi*) reduced lifespan similar to knockdown, which covered both developmental and adult stages (*elav-Gal4 > UAS-CactusRNAi*), indicating an adult-specific effect of Toll activation in both males and females (Figure 3A, 3B). We then conditionally targeted IPCs and observed that adult stage-specific *Cactus* knockdown in IPCs (*tub-Gal80^ts^;dilp5-Gal4 > UAS-CactusRNAi*) was sufficient to remarkably reduce the lifespan of these flies (Figure 3C, 3D). Finally, we generated a conditional neuroblast-specific model (*tub-Gal80^ts^;wor-Gal4 > UAS-CactusRNAi*), and intriguingly found the effect of *Cactus* knockdown in neuroblasts on lifespan to be developmentally restricted (Figure 3E, 3F). Token together, Toll pathway activation influences aging at different stages of life depending on the types of neural cells involved, with greater influence in neuroblasts during development and in IPCs during adulthood.

### Toll/NF-κB loss-of-function throughout all neurons modestly increases lifespan

Because we observed that genetic models for Toll pathway activation in neurons reduce lifespan, we next examined if inhibiting Toll activation could be beneficial for lifespan extension. To do so, we utilized two models for Toll pathway inhibition: either through *Cactus* overexpression (*UAS-Cactus*) or *Dif* knockdown (*UAS-DifRNAi*). These models represented two different genetic backgrounds with *UAS-Cactus* backcrossed into *w^1118^* background and *UAS-DifRNAi* crosses in a mixed genetic background, with the genetically-matched *UAS* flies crossed to *w^1118^* flies as controls in each case. Using these models, we found that pan-neuronal *Cactus* overexpression (*elav-Gal4 > UAS-Cactus*) or *Dif* knockdown (*elav-Gal4 > UAS-DifRNAi*) led to small although statistically significant increase in lifespan compared to control flies (*w^1118^ > UAS-Cactus* and *w^1118^ > UAS-DifRNAi*) (Supplementary Figure 4A, 4C, 4E, 4G). We examined the body weight of these flies and confirmed that this was not due to difference in size (Supplementary Figure 4B, 4D, 4F, 4H). Thus, pan-neuronal Toll pathway inhibition does not substantially increase lifespan, possibly due to confounding consequences because the Toll pathway is compromised in all neurons, which might have negative impacts on certain physiology which could offset the potential longevity effect of *Drosophila* NF-κB inhibition.

### Strong longevity by co-inhibiting Toll/NF-κB restrictedly in IPCs and neuroblasts

**Figure 4:**
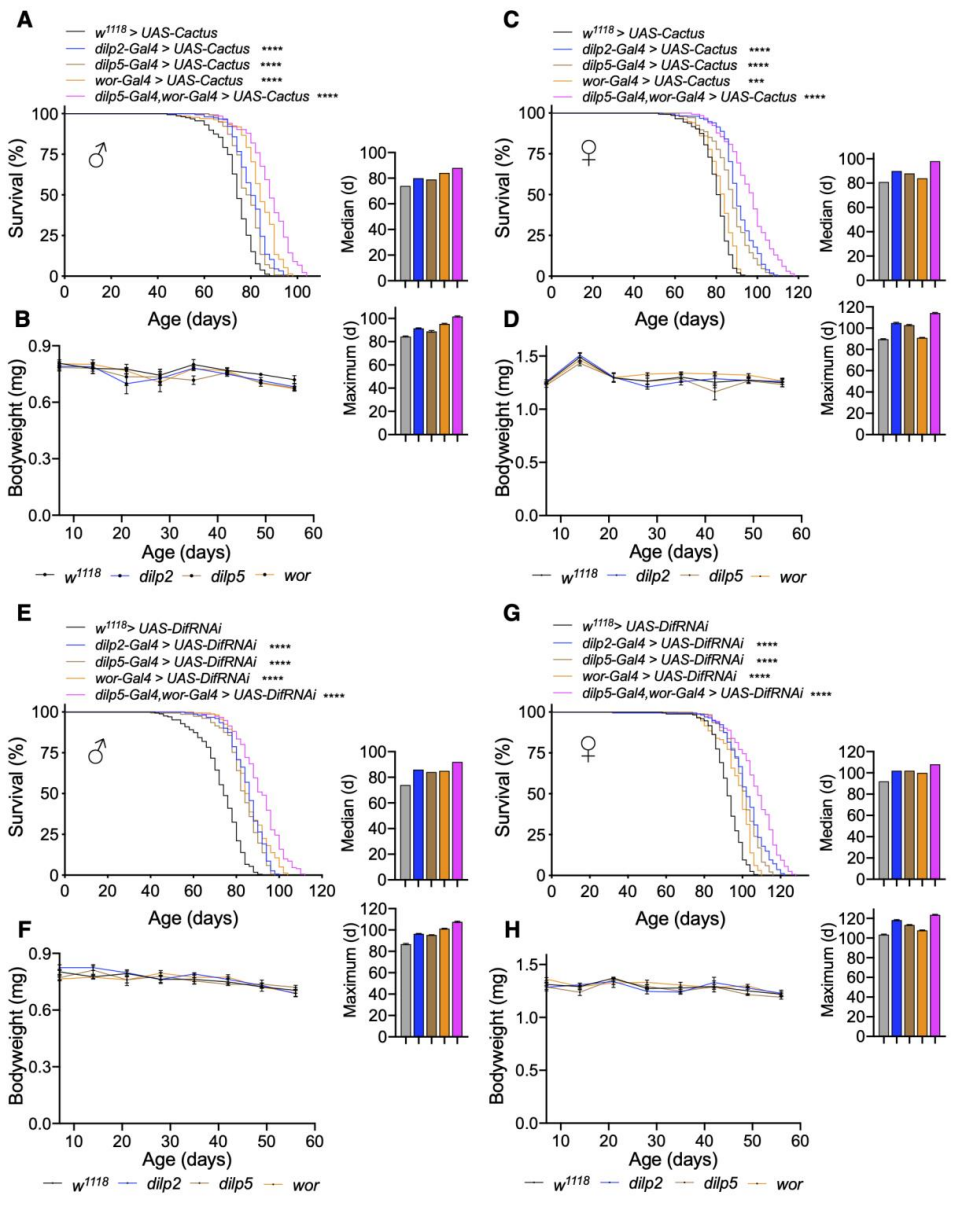
**Inhibition of Toll pathway signaling in IPCs or neuroblasts increases lifespan.** (**A**–**D**) Survival, median lifespan, and 10% max lifespan (**A**) and bodyweight (**B**) of *dilp2-Gal4/UAS-Cactus* males (blue), *dilp5-Gal4/UAS-Cactus* males (brown), *wor-Gal4/UAS-Cactus* males (orange), *dilp5-Gal4,wor-Gal4/UAS-Cactus* males (magenta), and *UAS-Cactus/+* (black) control males. Corresponding lifespan analysis (**C**) and bodyweight (**D**) for females. (**E**–**H**) Survival, median lifespan, and 10% max lifespan (**E**) and bodyweight (**F**) of *dilp2-Gal4/+;UAS-DifRNAi*/+ males (blue), *dilp5-Gal4/+;UAS-DifRNAi/+* males (brown), *wor-Gal4/+;UAS-DifRNAi/+* males (orange), *dilp5-Gal4,wor-Gal4/+;UAS-DifRNAi/+* males (magenta), and *+/+;UAS-DifRNAi/+* (black) control males. Corresponding lifespan analysis (**G**) and bodyweight (**H**) for females. Data information: statistics for curve comparisons are shown in the figure. Error bars represent mean ± SEM. *** p<.001, **** p<.0001 (log-rank test). n = at least 100 flies for each genotype in lifespan experiments. n = 4 vials of 15 flies for each genotype for bodyweight measurements.

As discussed above, pan-neuronal Toll inhibition could have widespread effects on nervous system physiology and function especially given the role of Toll signaling during development. In light of this, we sought to investigate more restrictive models by targeting only IPCs and neuroblasts. In doing so, we overexpressed *Cactus* in IPCs (*dilp2-Gal4 > UAS-Cactus* and *dilp5-Gal4 > UAS-Cactus*) or neuroblasts (*wor-Gal4 > UAS-Cactus*) and observed that *Cactus* overexpression in IPCs increased lifespan of females more than males (median lifespan increase of females of *dilp2-Gal4* and *dilp5-Gal4* crosses of 11.1% and 8.6% and maximum lifespan increase of 16.7% and 14.4% compared to median lifespan increase of 8.1% and 6.8% and maximum lifespan increase of 8.2% and 4.7% in males compared to *w^1118^ > UAS-Cactus* control) (Figure 4A, 4C). This is agreeable with prior observations that have observed that IPC ablation influences female lifespan more so than males [5]. We also measured fly body weight and did not observe a significant difference over the course of their lifespan (Figure 4B, 4D); thus, lifespan extension in these models was not associated with changes in body size. Targeted *Cactus* overexpression in neuroblasts extended the lifespan of males much more than females (median lifespan increase of 13.5% in males compared to 3.7% in females, maximum lifespan increase of 11.8% in males compared to 1.1% in females) (Figure 4A, 4C), which was in agreement with our observations that *Cactus* knockdown in neuroblasts reduced male lifespan more so than females (Figure 2A, 2C). We further verified an increase in the healthspan of these flies by assaying their negative geotaxis, which performed better than controls up to 70 days (Supplementary Figure 5A). Since insulin signaling could contribute to the lifespan increase, we measured expression of IPC *dilps* but did not see appreciable changes in their expression except a mild increase in *dilp5* expression (Supplementary Figure 5B). To further verify Toll pathway inhibition as the causative factor of the lifespan increase, we assayed *Dif* knockdown in IPCs or neuroblasts, which also led to lifespan extension although more equally between males and females (median lifespan increase of *dilp2-Gal4*, *dilp5-Gal4*, or *wor-Gal4* crosses of 16.2%, 13.5%, 14.9% in males compared to 10.9%, 10.9%, 8.7% in females, maximum lifespan increase of 10.3%, 9.2%, and 16.1% in males compared to 14.5%, 9.7%, 4.9% in females) (Figure 4E, 4G). Consistently, we did not observe changes in body weight for both sexes (Figure 4F, 4H). Thus, converse to Toll pathway gain-of-function models in IPCs or neuroblasts leading to lifespan loss (Figure 2), loss-of-function models led to lifespan extension apparently due to an anti-aging effect. Because these experiments indicated that Toll pathway inhibition in IPCs or neuroblasts could increase fly lifespan, we hypothesized whether targeting the Toll pathway in both cell types together might further increase lifespan. Indeed, we found that blocking Toll signaling in both cell types through *Cactus* overexpression increased lifespan more than the single drivers did (median lifespan increase of *dilp5-Gal4,wor-Gal4 > UAS-Cactus* males and females of 18.9% and 18.5%, maximum lifespan increase of 20% and 26.7%) (Figure 4A, 4C). We similarly observed a substantial increase in lifespan with *Dif* knockdown as well (median lifespan increase of *dilp5-Gal4,wor-Gal4 > UAS-DifRNAi* males and females of 24.3% and 17.4%, maximum lifespan increase of 24.4% and 20.4%) (Figure 4E, 4G). Taken together, Toll signaling within IPCs and neuroblasts is critical for fly aging, and combinatorial inhibition of both cell types can lead to a great increase in lifespan including maximum lifespan extension.

### Lifespan gain by inhibiting Toll/NF-κB in different cell types and different stages

**Figure 5:**
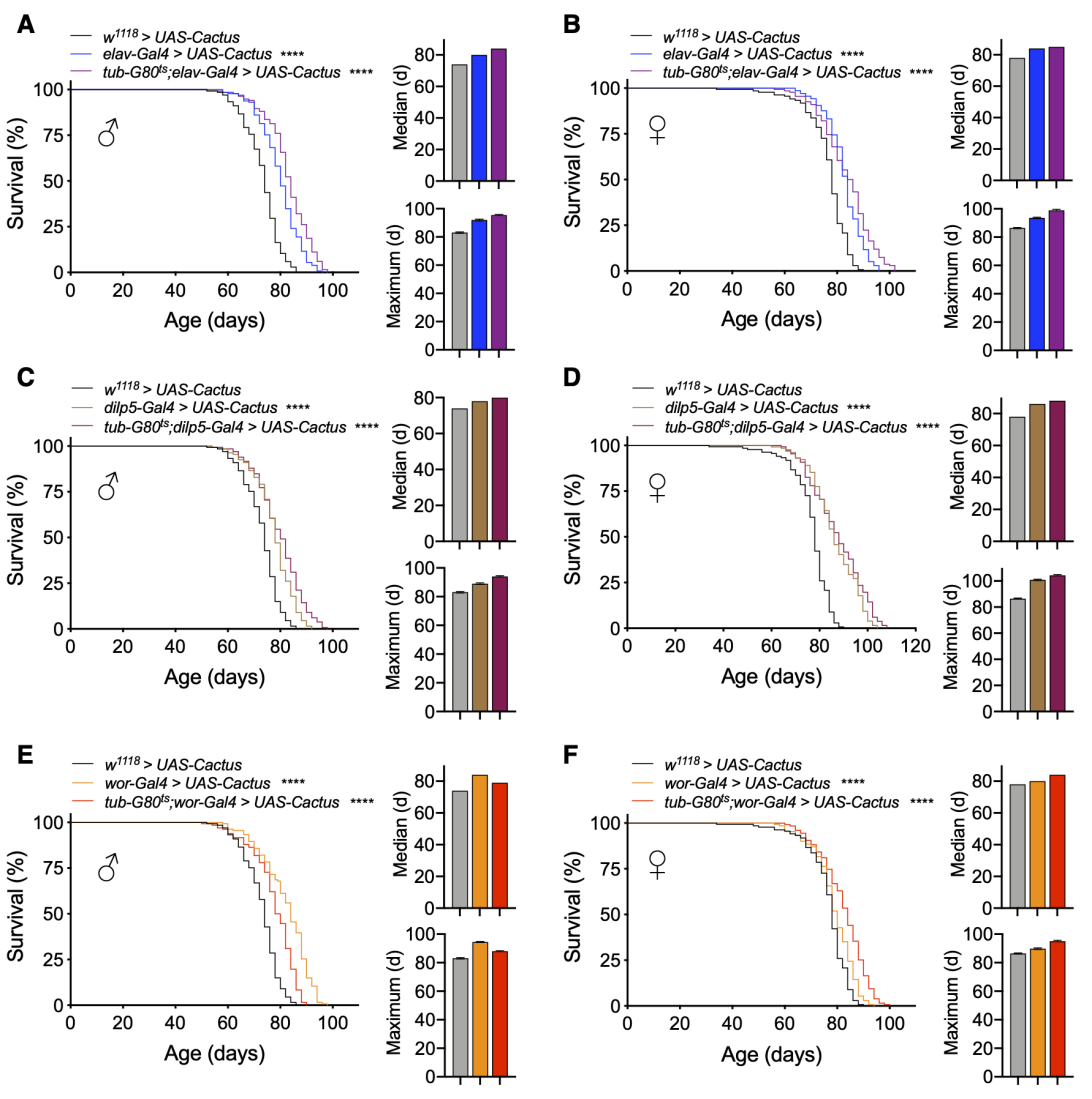
**Lifespan effects of Toll pathway inhibition in IPCs and neuroblasts are stage-dependent.** (**A**, **B**) Survival, median lifespan, and 10% max lifespan of *UAS-Cactus/+;elav-Gal4/+* males (blue), *tub-Gal80^ts^/UAS-Cactus;elav-Gal4/+* males (purple), and *UAS-Cactus/+;+/+* (black) control males (**A**). Corresponding lifespan analysis for females (**B**). (**C**, **D**) Survival, median lifespan, and 10% max lifespan of *dilp5-Gal4/UAS-Cactus;+/+* males (brown), *dilp5-Gal4/UAS-Cactus;tub-Gal80^ts^/+* males (purple), and *UAS-Cactus/+;+/+* (black) control males (**C**). Corresponding lifespan analysis for females (**D**). (**E**, **F**) Survival, median lifespan, and 10% max lifespan of *wor-Gal4/UAS-Cactus;+/+* males (orange), *wor-Gal4/UAS-Cactus;tub-Gal80^ts^/+* males (red), and *UAS-Cactus/+;+/+* (black) control males (**E**). Corresponding lifespan analysis for females (**F**). Data information: statistics for curve comparisons are shown in the figure. Error bars represent mean ± SEM. **** p<.0001 (log-rank test). n = at least 100 flies for each genotype in lifespan experiments.

Given that we had observed neural Toll pathway gain-of-function models through *Cactus* knockdown to influence lifespan in a stage-specific manner, we similarly employed conditional genetic models for Toll pathway loss-of-function. Indeed, we found that adult-specific pan-neuronal *Cactus* overexpression (*tub-Gal80^ts^;elav-Gal4 > UAS-Cactus*) increased lifespan, even slightly more than our non-conditional overexpression model, which covered both the development stage and adulthood (median lifespan increase with adult-specific versus development + adulthood expression of 13.5% and 8.1% compared to control for males and 9% and 7.7% for females, maximum lifespan increase of 14.5% and 10.8% for males and 15.1% and 9.3% for females) (Figure 5A, 5B). This difference suggested that the Toll signaling pathway has a role in the development of the nervous system and inhibiting it may have a negative impact on health and thus lifespan.

Focusing on our cell type-specific models, we observed that adult-specific *Cactus* overexpression in IPCs (*tub-Gal80^ts^;dilp5-Gal4 > UAS-Cactus*) recapitulated our findings from the models in which *Cactus* overexpression occurred during both development and adulthood (median lifespan increase compared to control with adult-specific expression versus development + adulthood expression of 8.1% compared to 5.4% control for males and 12.8% and 10.2% for females, maximum lifespan increase compared to control of 13.2% and 7.2% for males and 20.9% and 17.4% for females) (Figure 5C, 5D). Finally, we observed that the sex-specific male lifespan increase observed with *Cactus* overexpression in neuroblasts was due to developmental effects with a partial reduction in the extent of lifespan increase compared to non-conditional models. Intriguingly, female lifespan was slightly increased with this model, possibly suggesting there could be a benefit in adulthood for females (median lifespan increase compared to control with adult-specific expression versus development + adulthood expression of 6.7% compared to 13.5% control for males and 7.7% and 2.5% for females, maximum lifespan increase compared to control of 6% and 13.2% for males and 10.5% and 4.6% for females) (Figure 5E, 5F). These results together suggest a consistent effect, which mirrors what was seen in our conditional gain-of-function models that the Toll pathway influences aging differentially between IPCs and neuroblasts, being more important developmentally for neuroblasts and in adulthood for IPCs.

### Toll/NF-κB inhibition in IPCs leads to enhanced survival in various fatal stresses

**Figure 6:**
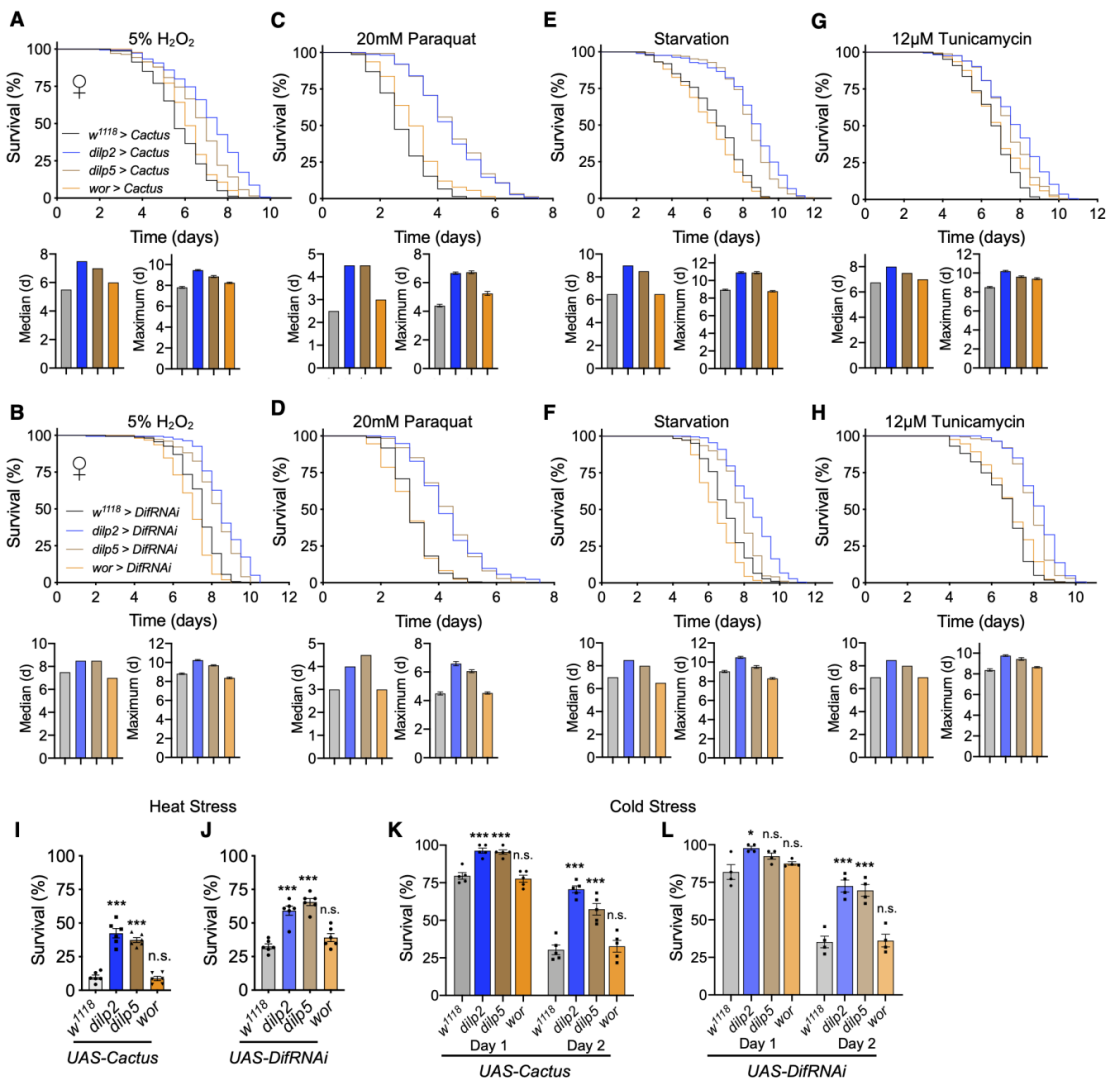
**Inhibition of Toll pathway signaling in IPCs improves survival in stress conditions.** (**A**, **C**, **E**, **G**) Survival, median survival, and 10% max survival of *dilp2-Gal4/UAS-Cactus* females (blue), *dilp5-Gal4/UAS-Cactus* females (brown), *wor-Gal4/UAS-Cactus* females (orange), and *UAS-Cactus/+* (black) control females exposed to 5% H_2_O_2_ (**A**), 20mM paraquat (**C**), starvation (**E**), or 12μM tunicamycin (**G**). (**B**, **D**, **F**, **H**) Survival, median survival, and 10% max survival of *dilp2-Gal4/+;UAS-DifRNAi/+* females (blue), *dilp5-Gal4/+;UAS-DifRNAi/+* females (brown), *wor-Gal4/+;UAS-DifRNAi/+* females (orange), and *+/+;UAS-DifRNAi/+* (black) control females exposed to 5% H_2_O_2_ (**B**), 20mM paraquat (**D**), starvation (**F**), or 12μM tunicamycin (**H**). (**I**, **J**) 24 hr survival of *UAS-Cactus* crosses (**I**) or *UAS-DifRNAi* crosses (**J**) outlined above after exposure to 37° C heat stress. (**K**, **L**) 24 and 48 hr survival of *UAS-Cactus* crosses (**K**) or *UAS-DifRNAi* crosses (**L**) outlined above after exposure to 0° C cold stress and allowed to recover for 24 or 48hr. Data information: statistics for curve comparisons are shown in the figure. Error bars represent mean ± SEM. * p<.05, *** p<.001, n.s. not significant (Student’s t-test). n = at least 100 flies for each genotype in stress assays (**A**–**H**). n = 4-5 vials of 20 flies for each genotype in temperature stress assays (**I**–**L**).

Stress resistance is commonly associated with various models of increased healthspan [5, 38]. Since we observed that Toll pathway inhibition in IPCs increased fly lifespan and healthspan, we investigated if it also would confer resistance to a variety of stressors leading to increased survival. To do this, we exposed flies to various established fatal conditions of stress including oxidative stress, starvation, endoplasmic reticulum (ER) stress, and temperature stress. Using our models of *Cactus* overexpression or *Dif* knockdown in IPCs or neuroblasts, we examined the response to oxidative stress through 2 methods: exposure to hydrogen peroxide (H_2_O_2_) or paraquat, a known oxidative stress inducer [39]. We found that Toll pathway inhibition in IPCs through either model increased stress resistance to either peroxide or paraquat treatment, more strongly so with paraquat treatment (Figure 6A–6D). However, overexpression in neuroblasts did not offer an appreciable benefit of stress resistance (Figure 6A–6D). We next exposed flies to starvation and ER stress and observed similar patterns in these conditions (Figure 6E–6H). To evaluate temperature stress, we examined fly recovery from exposure to acute heat stress or cold stress and observed similarly that Toll pathway inhibition in IPCs but not neuroblasts enhanced survival to temperature stress (Figure 6I–6L). Given that we tested various stress induction mechanisms, the battery of these results suggested that the lifespan effect of Toll pathway in IPCs involves a control over stress, while the lifespan effect of Toll pathway in neuroblasts seems to be independent of this physiology.

## DISCUSSION

The process of aging is regulated by many intrinsic factors within an organism, including inflammatory reactions governed by innate immune activation. The signaling mechanisms at play that underlie this process include the activation of NF-κB signaling leading to the production of inflammatory insults that are pro-aging. Recent research has brought to light the significance of neural inflammatory signaling as a component of regulation over systemic aging. In this study, we sought to characterize whether NF-κB signaling in Toll versus Imd pathways in *Drosophila* are equally influential to control of aging and lifespan. We found that neuronal activation of either Toll or Imd signaling decreases fly lifespan, and comparatively, the Toll pathway is more sensitively involved. Furthermore, we found that neuroendocrine IPCs or neuroblasts are important for these effects with a stronger phenotype observed with Toll activation. This information suggests a level of programmatic control over aging, given that this expression is restricted to a rather small population of cells in the fly brain. We conversely observed Toll/NF-κB inhibition to increase fly lifespan and healthspan, and co-inhibition in IPCs and neuroblasts could increase lifespan more than either alone, suggesting that these two cell types might have independent mechanisms in modulating lifespan.

Prior work in mice found increased NF-κB activity in the brains of middle-aged and older mice, and brains of aged *Drosophila* were shown to have increased AMP expression, indicative of increased innate immune signaling [6, 30]. We observed that increasing Toll or Imd activity in neurons decreased fly lifespan, supporting increased neuronal NF-κB activity as being important to aging. On the other hand, our models for pan-neuronal Toll pathway inhibition only modestly increased lifespan. Thus, while NF-κB activation in all neurons provides a tremendous contribution to aging and lifespan limit, utilizing this approach for NF-κB inhibition to obtain the benefit of anti-aging and lifespan extension is not ideal. This is logical because NF-κB genes are probably expressed in all cells of the nervous system and could be biologically necessary during certain conditions such as the developmental stage or reactions to environmental challenges. Moreover, it is likely that decreasing the expression of these genes might negatively affect some neurons or neural circuities. For this reason, we focused our effort toward understanding how Toll or Imd gain-of-function models influence lifespan in restricted subsets of cells within the nervous system. The IPCs of the pars intercerebralis were of particular interest to us, given their homology to the mammalian hypothalamus. Rodent models observed NF-κB activity to change with aging most drastically within the hypothalamus [6], which provoked us to examine if this could hold true for *Drosophila* IPCs. In the literature, reduction of insulin signaling has been associated with longevity, and fly models for IPC ablation or ablation of the various Dilp peptides produced by IPCs have been shown to increase healthspan and longevity [5, 12]. Our observations that Toll/NF-κB gain-of-function or loss-of-function in IPCs decreases and increases lifespan, respectively, suggests that these cells are sensitive to innate immune signaling for lifespan effects. Expression levels of dilps in these models were either unchanged or changed without favoring the lifespan phenotypes, suggesting that the Toll pathway may modulate other neurohormonal mechanisms in these cells to affect lifespan.

Apart from IPCs, we were interested to examine the effects of Toll and Imd pathway manipulation in neuroblasts. The role of these cells in *Drosophila* aging has not been appreciated to date, which may be due to the difficulty of detecting adult neuroblasts. However, a recent study in *Drosophila* did find residual neuroblast populations capable of neurogenesis in response to traumatic injury, suggesting that there may be some low level of adult neurogenesis [20]. Our conditional model for adult-specific changes in Toll signaling in neuroblasts could not fully recapitulate the changes in lifespan in our models encompassing development, suggesting that these cells are more developmentally restricted. In addition to neurogenesis, Toll signaling in neuroblasts could affect structure and functions of existing neurons which might be important for regulate lifespan. However, we could identify a modest effect on lifespan due to manipulation of the Toll pathway during the adulthood, which indirectly supports a limited existence of these cells during the adult stage. Comparatively, hypothalamic stem cells were found to be crucial for mammalian aging [7], suggesting that the role of neural stem cells for aging is more important in more advanced species. Given that Toll inhibition within IPCs or neuroblasts separately led to increased lifespan, we examined the efficacy of a model of dual inhibition. This was especially supported by our observed importance of neuroblast Toll signaling to lifespan changes during development and IPC Toll signaling during adulthood. We observed an additive effect when combining these two models of Toll pathway inhibition, which suggested that they utilize some different mechanisms to regulate aging progression. Additionally, it supports the notion that rather than targeting innate immune changes in individual groups of cells in the nervous system, it may be more efficacious to downregulate such processes in multiple subpopulations. Given that lineage tracing revealed a single pair of neuroblasts gives rise to IPCs during development [40], it is possible that neuroblast Toll inhibition could lead to a change in IPCs that later becomes beneficial against aging, but this warrants further investigation.

An appreciation for the role of neural innate immune signaling mechanisms in aging progression is slowly emerging. Recent studies in rodents and *Drosophila* have linked changes in neural NF-κB signaling to aging and neurodegenerative disease by generally targeting neurons and glial populations [6, 30–32]. In this study, we attempted to refine these models and better dissect how innate immune activity in subpopulations of cells within the nervous system contributes to aging. We additionally identified that these changes have differential effects in targeting either the Toll or Imd pathway, are sensitively dependent on the stage of the fly lifecycle, and when combined can further increase lifespan. As our understanding of neuroinflammatory mechanisms increases, it will become clearer how changes in relatively small populations of cells can influence aging progression. The translation of these findings from rodents to *Drosophila* indicates an evolutionary preservation of the contribution of neural innate immune signaling mechanisms in aging progression and lifespan control.

## MATERIALS AND METHODS

### Fly strains

The following fly stocks were all obtained from the Bloomington Drosophila Stock Center: *w^1118^* (no. 5905), *UAS-CactusRNAi* (no. 34775), *UAS-DifRNAi* (no. 30513), *UAS-Relish* (no. 9459), *UAS-Rel* (no. 55778), *elav-Gal4* (no. 8760), *dilp2-Gal4* (no. 37516), *dilp5-Gal4* (no. 66007), *wor-Gal4* (no. 56553), *Sna^Sco^/CyO;tub-Gal80^ts^* (no. 7018), and *tub-Gal80^ts^;TM2/TM6B* (no. 7108). *UAS-Dif* [41] was a gift from Tony Ip (University of Massachusetts Medical School) and *UAS-Cactus* [42] was a gift from Shubha Govind (City University of New York). All Gal4 strains, *UAS-Relish*, *UAS-Dif*, and *UAS-Cactus* were backcrossed to *w^1118^* control line at least 10 times. Flies were housed at 25° C and 60% relative humidity under a 12:12-h light/dark cycle.

### Lifespan analysis

For all experiments, virgin female *UAS* and male *Gal4* or *w^1118^* control flies were bred together. Heterozygous controls were obtained by crossing *UAS* effectors to *w^1118^*. Newly eclosed flies were collected over 2-day periods and transferred to 10% sugar/yeast (10% S/Y) media and allowed to mate for 48 hours before sorting males and females. For experiments using *Gal80^ts^*, flies were bred at the permissive temperature (18° C) during developmental stages, and newly eclosed flies transferred to the non-permissive temperature (25° C) for the duration of their adult lifespan. Replicate density for all experiments was set to about 20 flies per vial. Flies were switched to fresh food every 2 days and mortality was recorded. Each experiment was performed with 3-4 biological replicates per group and repeated at least twice.

### Body weight measurement

For bodyweight measurement, flies were kept at a density of 15 flies per vial. Flies were briefly anesthetized on ice, transferred to empty vials, and weight was recorded. 3-4 replicates were used for each group.

### Negative geotaxis

Negative geotaxis was performed by placing flies into empty vials divided into 3 quadrants: upper 1/3, middle 1/3, and lower 1/3. Vials were placed in a geotaxic apparatus. Flies were tapped to the bottom and allowed to climb vials for 10 or 20 seconds after which the number of flies in each quadrant was recorded. This was repeated three times with one minute rest periods between each technical replicate.

### Quantitative PCR

Total RNA was extracted from 60 *Drosophila* heads for each sample and RNA was isolated using Direct-zol RNA MiniPrep (Zymo Research) according to the manufacturer’s instructions. mRNA was transcribed to cDNA using the M-MLV RT System (Promega). Gene expression was analyzed using SYBR Green PCR Master Mix (Applied Biosystem) and normalized to expression levels of rp49. Primer sequences are as follows: dilp2 F: 5’- gaatcacgggattatactcctcg-3’, dilp2 R: 5’- atgagcaagcctttgtccttca-3’, dilp5 F: 5’- gaggcaccttgggcctattc-3’, dilp5 R: 5’-catgtggtgagattcggagcta-3’, rp49 F: 5’-ccgcttcaagggacagtatc-3’, and rp49 R: 5’-gacaatctccttgcgcttct-3’.

### Stress assays

Newly eclosed males and females were collected over 2-day periods and transferred to 10% sugar/yeast (10% S/Y) media and allowed to mate for 48 hours before sorting females. Flies were aged to 10 days before exposing to stressors. Mortality was recorded every 12 hours following transfer to stress conditions. Replicate density for all experiments was set to about 20 flies per vial. Each stress assay was performed with 3-4 biological replicates per group and repeated at least twice. For oxidative stress assays, 10-day old flies were transferred to vials containing 1.5% agar, 5% sucrose, 5% H_2_O_2_ (Acros Organics, AC302865000) for peroxide treatment, or 1.5% agar, 5% sucrose, 20mM paraquat (Acros Organics, AC227320010) for paraquat treatment. For metabolic stress assays, 10-day old flies were transferred to vials containing 1.5% agar dissolved in water. For ER stress assays, 10-day old flies were transferred to vials containing 1.5% agar, 5% sucrose, 12 μM tunicamycin (Sigma, T7765). For heat stress assays, 10-day old flies were transferred to empty vials and submerged in a 37° C water bath for 4 hours (*UAS-Cactus*) or 6.5 hours (*UAS-DifRNAi*). Flies were allowed to recover for 24 hours and assessed for mortality. For chill coma recovery assays, 10-day old flies were submerged in an ice bath at 4° C for 20 hours and allowed to recover for 24 and 48 hours and assessed for mortality.

### Statistical analysis

GraphPad Prism software (GraphPad) was used for all statistical analysis. Survival curves of different genotypes were analyzed using log rank test. Maximum lifespan was calculated from the top 10% of each cohort. Stress assay comparisons were made using student’s t-test. In all tests, p < 0.05 was considered significant.

## Supplementary Material

Supplementary Figures
