# Supplementary material for: Control of lifespan and survival by Drosophila NF-κB signaling through neuroendocrine cells and neuroblasts
Source: Aging (Albany NY). 2020 Nov 24;12(24):24604–22. doi: 10.18632/aging.104196 (PMC7803524; doi:10.18632/aging.104196)
Supplement: Supplementary Figures [file aging-12-104196-s001.pdf]

Supplementary Figures

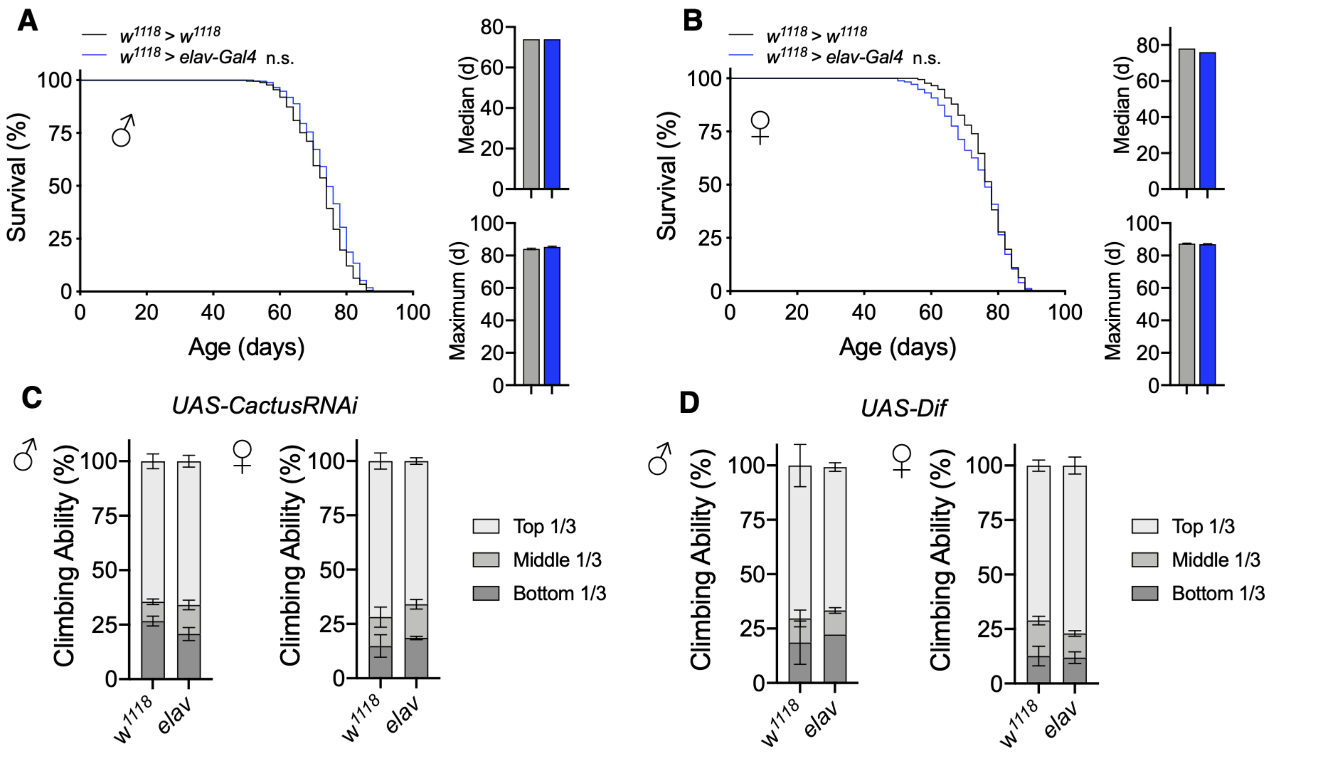

Supplementary Figure 1. *elav-Gal4* does not affect lifespan. (**A**, **B**) Survival, median lifespan, and 10% max lifespan of *w*^{1118}*;;elav-Gal4/+* males (blue) and *w*^{1118}*;;+/+* (black) control males (**A**). Corresponding lifespan analysis for females (**B**). (**C**) 10-day negative geotaxis for *elav-Gal4/UAS-CactusRNAi* and *UAS-CactusRNAi/+* females. (**D**) 10-day negative geotaxis for *elav-Gal4/UAS-Dif* and *UAS-Dif/+* females. Data information: statistics for curve comparisons are shown in the figure. Error bars represent mean ± s.e.m. n.s. not significant (log-rank test).

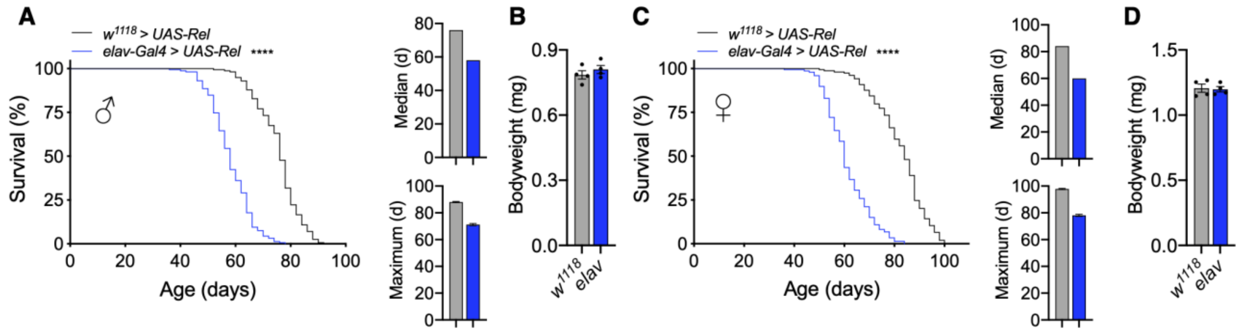

Supplementary Figure 2. Pan-neuronal constitutive Imd pathway activation shortens lifespan. (**A**–**D**) Survival, median lifespan, and 10% max lifespan (**A**) and bodyweight (**B**) of *elav-Gal4/UAS-Rel* males (blue) and *UAS-Rel/+* (black) control males. Corresponding lifespan analysis (**C**) and bodyweight (**D**) for females. Data information: statistics for curve comparisons are shown in the figure. Error bars represent mean ± s.e.m. **** p<.0001 (log-rank test).

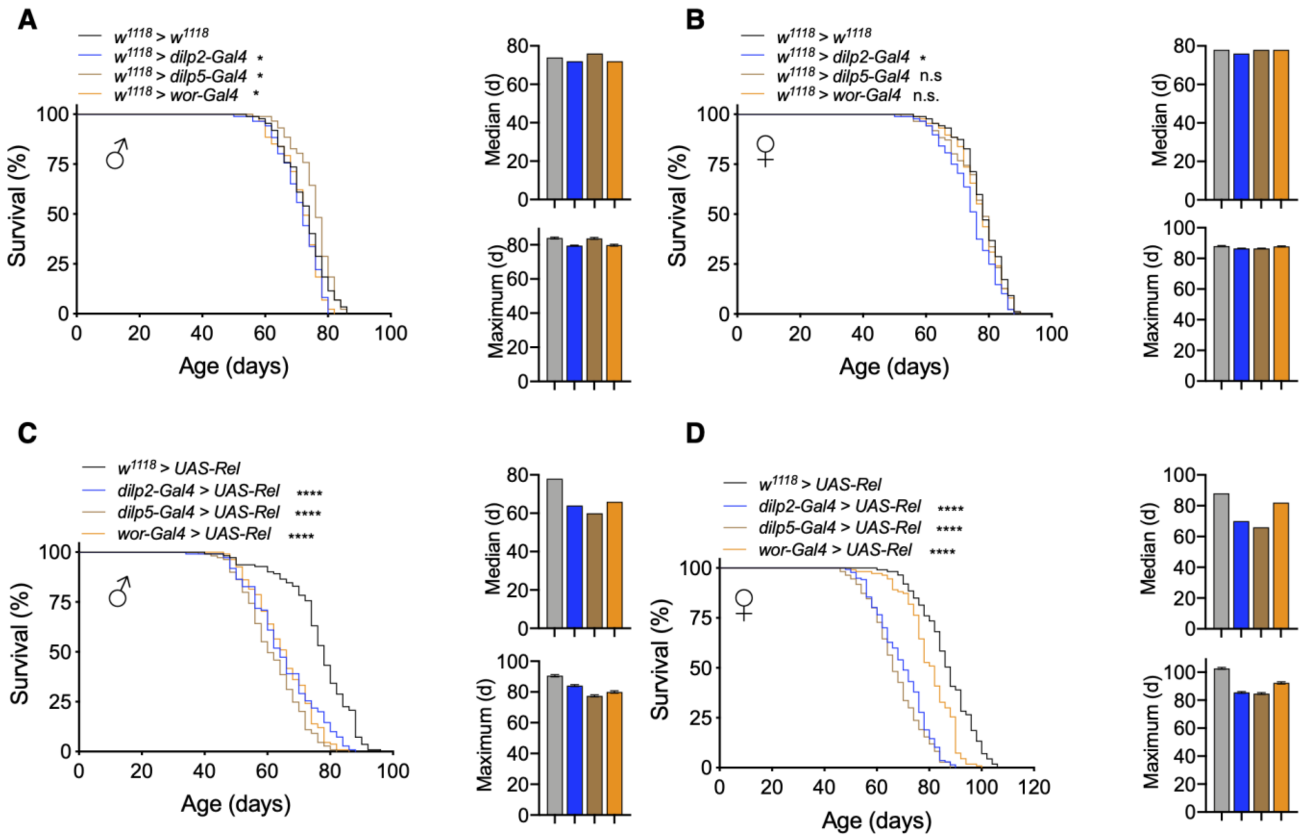

Supplementary Figure 3. Constitutive Imd pathway activation in IPCs or neuroblasts shortens lifespan. (**A**, **B**) Survival, median lifespan, and 10% max lifespan of *w*^{1118}*;dilp2-Gal4/+* males (blue), *w*^{1118}*;dilp5-Gal4/+* males (brown), *w*^{1118}*;wor-Gal4/+* males (orange), and *w*^{1118}*;+/+* (black) control males (**A**). Corresponding lifespan analysis for females (**B**). (**C**, **D**) Survival, median lifespan, and 10% max lifespan of *dilp2-Gal4/UAS-Rel* males (blue), *dilp5-Gal4/UAS-Rel* males (orange), *wor-Gal4/UAS-Rel* males (brown), and *UAS- Rel/+* (black) control males (**C**). Corresponding lifespan analysis for females (**D**). Data information: statistics for curve comparisons are shown in the figure. Error bars represent mean ± s.e.m., n.s. not significant, * p<.05, **** p<.0001 (log-rank test).

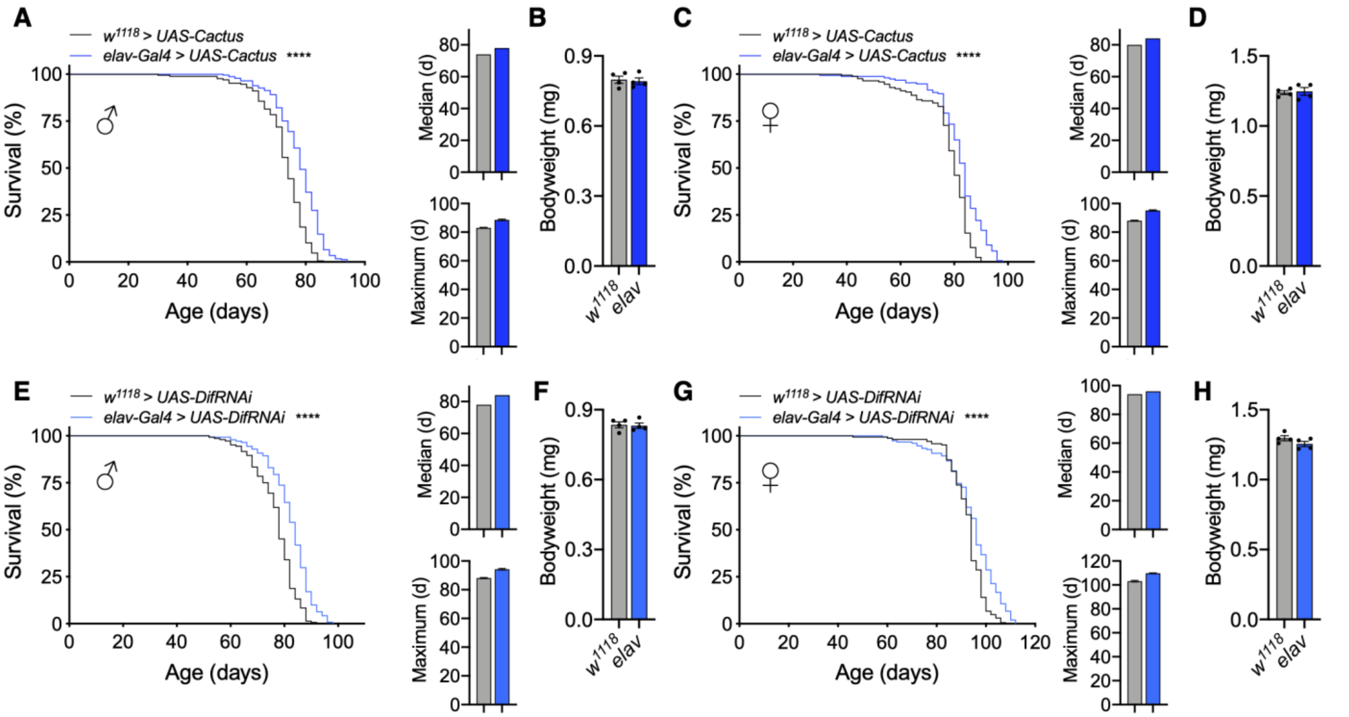

Supplementary Figure 4. Pan-neuronal Toll pathway inhibition modestly increases lifespan. (**A**, **D**) Survival, median lifespan, and 10% max lifespan (**A**) and bodyweight (**B**) of *UAS-Cactus/+;elav-Gal4/+* males (blue) and *UAS-Cactus/+;+/+* (black) control males. Corresponding lifespan analysis (**C**) and bodyweight (**D**) for females. (**E**–**H**) Survival, median lifespan, and 10% max lifespan (**E**) and bodyweight (**F**) of *UAS-DifRNAi/elav-Gal4* males (blue) and *UAS-DifRNAi/+* (black) control males. Corresponding lifespan analysis (**G**) and bodyweight (**H**) for females. Data information: statistics for curve comparisons are shown in the figure. Error bars represent mean ± s.e.m. **** p<.0001 (log-rank test).

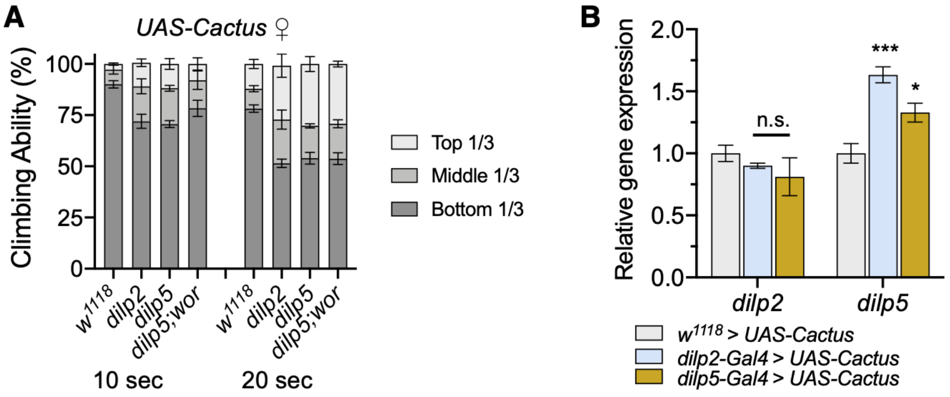

Supplementary Figure 5. Toll pathway inhibition in IPCs increases climbing ability. (**A**) 70-day negative geotaxis for *dilp2-Gal4/UAS-Cactus*, *dilp5-Gal4/UAS-Cactus*, *dilp5-Gal4,wor-Gal4/UAS-Cactus*, and *UAS-Cactus/+* females. (**B**) Gene expression for *dilp2* and *dilp5* in flies with Cactus overexpression in IPCs versus controls (n = 4). Data information: statistics for curve comparisons are shown in the figure. Error bars represent mean ± s.e.m. n.s., not significant, * p<.01, *** p<.001.
